# Functional morphology of cleaning devices in the damselfly *Ischnura elegans* (Odonata, Coenagrionidae)

**DOI:** 10.3762/bjnano.15.102

**Published:** 2024-10-16

**Authors:** Silvana Piersanti, Gianandrea Salerno, Wencke Krings, Stanislav Gorb, Manuela Rebora

**Affiliations:** 1 Dipartimento di Chimica, Biologia e Biotecnologie, University of Perugia, Via Elce di Sotto 8, 06121 Perugia, Italy; 2 Dipartimento di Scienze Agrarie, Alimentari e Ambientali, University of Perugia, Borgo XX Giugno, 06121 Perugia, Italy; 3 Department of Cariology, Endodontology and Periodontology, Universität Leipzig, Liebigstraße 12, 04103 Leipzig, Germany; 4 Department of Functional Morphology and Biomechanics, Zoological Institute, Kiel University, Am Botanischen Garten 1–9, 24098 Kiel, Germany

**Keywords:** antennae, cuticle, eyes, grooming, legs, resilin

## Abstract

Among the different micro- and nanostructures located on cuticular surfaces, grooming devices represent fundamental tools for insect survival. The present study describes the grooming microstructures of the damselfly *Ischnura elegans* (Odonata, Coenagrionidae) at the adult stage. These structures, situated on the foreleg tibiae, were observed using scanning electron microscopy, and the presence and distribution of resilin, an elastomeric protein that enhances cuticle flexibility, were analyzed using confocal laser scanning microscopy. Eye and antennal grooming behavior were analyzed to evaluate the particle removal efficiency in intact insects and in insects with ablated grooming devices. The grooming devices are constituted of long setae from which a concave cuticular lamina develops towards the medial side of the leg. Each seta shows a material gradient of resilin from its basal to the distal portion and from the seta to the cuticular lamina. The removal of the grooming devices induces a strong increase in the contaminated areas on the eyes after grooming. Further studies on insect grooming can provide valuable data on the functional morphology of insect micro- and nanostructures and can represent a starting point to develop advanced biomimetic cleaning tools.

## Introduction

Self-grooming, defined as any behavior related to the maintenance and care of body surfaces, is an innate behavior found across a wide range of animal species, from vertebrates to arthropods, with early evolutionary origins (reviews in [1–2]). Despite the distant evolutionary relationship between vertebrates and insects, their grooming behaviors serve multiple and similar purposes, such as body cleaning and disease prevention, distribution of substances across the body surface, maintenance of sensory organs, and displacement behavior in stressful conditions [3].

In insects, the chitinous exoskeleton, with the epidermis below it, forms the integumentary boundary between internal organs and the external environment. The exoskeleton can perform numerous tasks through the presence of micro- and nanostructures located on its cuticular surface, serving functions from sensory reception to surface adhesion, air retention, food grinding, thermoregulation, and color production (reviews in [4–5]). The insect cuticle is frequently exposed to a variety of inorganic and organic particles, which can disrupt its normal function or hinder essential physiological processes, ultimately decreasing survival rates. As a result, insects dedicate a considerable amount of time to self-grooming to eliminate debris [6–7], parasites [8], and pathogens [9]. This grooming behavior also plays a role in distributing substances across their bodies, such as antimicrobial compounds [10], brochosomes [11], and cuticular lipids [6,12]. Additionally, self-grooming is essential for flight, as it keeps the wings clean [13], and for movement on land, by ensuring the cleanliness of the tarsi and maintaining the adhesion of attachment pads [14–16]. Cleaning behavior plays an exceptionally important role in social insects like ants, for example, to guarantee precise nestmate/non-nestmate discrimination [17] or in mutual grooming [18]. Additionally, grooming plays a pivotal role in maintaining olfactory acuity. Böröczky et al. [19] demonstrated that antennal grooming removes not only foreign chemicals but also excess native cuticular lipids that may interfere with olfaction, thereby maintaining the olfactory sensitivity of the antennae.

In insects, body cleaning devices are typically located on the legs and are associated with complex grooming behaviors that vary greatly across arthropods [20]. Numerous studies on flies [21–22], wasps [23], mantids [24], and crickets [25] indicate that grooming behavior often falls into two distinct clusters. The anterior cluster, predominantly using the forelegs, involves grooming the antennae, head, mesosoma, forelegs, and middle legs. The posterior cluster focuses on cleaning the wings, metasoma, middle legs, and hind legs, and primarily uses the hind legs. A similar behavior is reported in ants, where functional morphology and efficiency of the grooming activity have been largely investigated in old and recent papers [26–27].

The antenna cleaner is usually formed from a modified fore tibia, tibial spurs, and/or fore basitarsus, but its morphology varies greatly among groups [20]. In Hemiptera, antennal grooming involves scraping with the tibial comb complex (tibial comb + fossula) of both forelegs on the antenna, generally followed by grooming the tibial comb complex of one leg using the tarsal hairy pad of the opposite leg [28]. In Lepidoptera, many groups use a comb-like spur on the fore tibia for antennal cleaning [20,29]. In Diptera, tibial grooming combs are found on the ventral apices of the fore tibiae in mosquitoes [22]. In Hymenoptera, one of the fore tibial spurs, called calcar, is highly modified for antennal grooming, and usually the basitarsus is also specialized for this purpose; the two parts acting together form an anatomical cluster called strigil [26–27,30]. In Coleoptera the protibial antennal cleaning organ is the main argument in support of a clade Geadephaga [31].

Studies on insect grooming can provide valuable data on the functional morphology of insect micro- and nanostructures and can enhance our understanding of different insect behavior and evolution (e.g., [32] for Mantodea and [26] for Hymenoptera). Moreover, they can represent the starting point to develop useful biomimetic tools [33]. Studies on grooming devices in Paleoptera (Odonata and Ephemeroptera) are scarce. Except for an old description of odonatan forelegs by St. Quentin [34] and some scattered observations of the grooming behavior in some odonatan species (review in [35]), no detailed study has been performed so far.

This study aims to describe the grooming devices located on the forelegs of a damselfly that are used to clean the head and, especially, the eyes and the antennae. The microstructures were observed using scanning electron microscopy (SEM), and the presence and distribution of resilin, an elastomeric protein that enhances cuticle deformability and flexibility (review in [36]), were analyzed using confocal laser scanning microscopy (CLSM). The eye and antennal grooming behavior of the damselfly *Ischnura elegans* (Vander Linden, 1820) adults (Odonata, Coenagrionidae) was observed and analyzed to evaluate the particle removal efficiency in intact and ablated insects.

## Material and Methods

### Insects

*Ischnura elegans* males and females were collected in the field at Centro Ittiogenico del Trasimeno - Sant’Arcangelo (Perugia, Umbria region, Italy), in spring and summer 2023–2024. They were maintained for 2–3 days in a controlled climate chamber (14:10 light–dark rhythm, at a temperature of 25 ± 1 °C and relative humidity of 70 ± 10%) inside net cages (25 cm × 25 cm × 25 cm). *Drosophila melanogaster* flies were used to feed the damselflies. Adult insects of both sexes were used in the study.

### Light microscopy

To count the mean number of foretibial grooming structures in males and females, we anaesthetized 13 males and eleven females with carbon dioxide, dissected their forelegs and observed them under a stereomicroscope Leica MZ6 (Leica Microsystem GmbH, Wetzlar, Germany).

To obtain semithin sections, the tibiae of six insects were dissected under the stereomicroscope. Samples were then fixed for 3 h in 2.5% glutaraldehyde in sodium cacodylate buffer (Electron Microscopy Sciences, Hatfield, PA, USA) with a pH of 7.2, repeatedly rinsed in sodium cacodylate buffer and post-fixed for 1 h at 4 °C in 1% osmium tetroxide in sodium cacodylate buffer (Electron Microscopy Sciences). Fixed samples were repeatedly rinsed in the same buffer, dehydrated by using ascending ethanol concentrations, and finally embedded in an Epon–Araldite resin mixture (Sigma-Aldrich). Afterwards, semithin sections of the foretibial grooming structures were cut with a diamond knife using a Leica EM UC6 ultramicrotome, collected on glass slides, stained with 1% methylene blue with sodium borate, and observed and photographed using a KOPPACE microscope camera KP-2100 (KOPPACE, Kepuaisi Science Technology, Shenzhen, China) mounted on a light microscope Leica DMLB (Leica Microsystem GmbH, Wetzlar, Germany).

### Scanning electron microscopy

Foretibiae were dissected from anaesthetised specimens (ten males and ten females), fixed for 12 h in 2.5% glutaraldehyde in cacodylate buffer (Electron Microscopy Sciences) at pH 7.2, repeatedly rinsed in the same buffer and dehydrated using ascending ethanol gradients (20%, 50%, 70%, 80%, 95%, and 100%), followed by drying in an oven at 40 °C for three days. Foretibiae of intact and ablated insects used to evaluate the particle removal efficiency were carefully dissected under a stereomicroscope and then dried in an oven at 40 °C for three days. They were not fixed to avoid removing the talc powder present on the grooming structures. The specimens were deposited on aluminum stubs using double-sided adhesive tape. Before the analysis, the samples were sputter-coated with a thin layer of gold (8 nm) using a Q150 T ES (Quorum, Laughton, UK) for 30 s. The samples were then analyzed in a field-emission scanning electron microscope FE SEM LEO 1525 (ZEISS, Oberkochen, Germany) at 5 kV accelerating voltage.

### Confocal laser scanning microscopy

A CLSM-based method established by Michels and Gorb [37], to analyze material compositions and their gradients in arthropod cuticle by visualizing autofluorescence, was applied to the foretibial grooming structures. We interpreted the final images and described the material properties of the cuticle as follows: (1) Red areas are likely well-sclerotized, (2) green-to-yellow areas are less sclerotized in comparison to red ones and mechanically stable, but relatively flexible because of the lower degree of sclerotization, and (3) blue areas are rubber-like with a relatively high proportion of resilin-like proteins or unsclerotized chitin. This method has already been widely applied in the literature [37–40].

The insects to be observed were frozen in a conventional lab freezer (ca. −20 °C) for 10 min. The foretibiae were cut from males and females by a scalpel. The specimens were washed in 70% ethanol and then immersed in glycerine (≥99.5%, Carl Roth GmbH & Co. KG, Karlsruhe, Germany). After fixing the specimens in glycerine between a glass slide and a cover slip (Carl Roth GmbH & Co. KG, Karlsruhe, Germany) for ca. 2 h, we visualized them with the CLSM (Zeiss LSM 700, Carl Zeiss Microscopy, Jena, Germany). The CLSM was equipped with four lasers (laser lines: 405, 488, 555, and 639 nm) to excite the sample fluorescence subsequently. Four emission filters transmitting 420–480, ≥490, ≥560, and ≥640 nm were used to visualize different fluorescence emissions of the cuticle components. We have visualized the dorsal and ventral cuticle from the foretibiae of males and females.

### Behavior

To describe the grooming behavior, living individuals were observed and video-recorded using a high-speed camera DMK 37BUX287 720×540, 539 FPS, global shutter mounted on a Leica MZ6 (Leica Microsystem GmbH, Wetzlar, Germany) stereomicroscope at 500 FPS. To induce grooming behavior, the insect antennae or eyes were fully covered with pink talc powder (Holi Colors Italia, Calatafimi, Italy) containing irregularly shaped particles (34.5 ± 3.5 µm), previously used in experiments on other insects for the same purpose [28]. In detail, in each experiment, insect antennae or eyes were covered with the powder by gently inserting each antenna or each eye of the live insect into a pipette tip filled with the powder. Video recording began immediately after contamination and continued for 5 min after beginning of the grooming. To describe the grooming behavior, the following parameters and acts (and their duration and frequency) were recorded with the software Solomon coder [41]:

- right foreleg raising and scraping of the right eye, or of the right eye and of the dorsal side of the right antenna with the right tibia (R eye-ant);
- left foreleg raising and scraping of the left eye, or of the left eye and of the dorsal side of the left antenna with the left tibia (L eye-ant);
- right foreleg raising and scraping of the right antenna with the right tibia (R ant);
- left foreleg raising and scraping of the left antenna with the left tibia (L ant);
- head rotation (70–90°) (head rot);
- right tibia running through mouthparts (R tibia cleaning);
- left tibia running through mouthparts (L tibia cleaning); and
- resting between two bouts (resting).

The behavior of four insects with contaminated eyes for a total of 17 bouts and the behavior of four insects with contaminated antennae for a total of eight bouts was analyzed. The behavior recording was stopped when the insect resting time after one bout was longer than 120 s.

The video in Supporting Information File 1 was recorded using a KOPPACE microscope camera KP-2100 (KOPPACE, Kepuaisi Science Technology, Shenzhen, China) mounted on a Leica MZ6 stereomicroscope (Leica Microsystem GmbH, Wetzlar, Germany).

In the experiments with contaminated eyes and with contaminated antennae, in consideration that the data were not normally distributed (Shapiro–Wilk test), to compare the frequency and the duration of forelegs raising and scraping of the eye or of the eye plus the dorsal side of the antennae with the tibiae (Eye-ant), the Mann–Whitney rank sum test was used. The Student *t*-test for independent samples was used to compare the frequency and duration of the other recorded acts (Ant, Tibia cleaning, Head rot) in the experiments with contaminated eyes and with contaminated antennae.

### Role of the foretibial cleaning structures and particle removal efficiency

We evaluated particle removal efficiency in experiments with intact and ablated insects, with their foretibial grooming structures artificially removed. To prepare ablated insects, they were anesthetized with carbon dioxide for 60 s, and the tibial grooming structures of the forelegs were carefully cut off with a scalpel blade under the stereomicroscope. The insects were left to recover for 24 h before carrying out the experiments. In each experiment, with intact or ablated insects, antennae or eyes were covered with pink talc powder (Holi Colors Italia, Calatafimi, Italy) as above described. At the beginning of the experiment, just after contamination, and at the end, after 1 h of grooming for the eyes and 10 min of grooming for the antennae, the head of the test insect was observed and photographed. Images of the head and of the forelegs before and after grooming were taken in intact and ablated insects using a KOPPACE microscope camera KP-2100 (KOPPACE, Kepuaisi Science Technology, Shenzhen, China) mounted on a Leica MZ6 stereomicroscope (Leica Microsystem GmbH, Wetzlar, Germany). In some specimens, mouthparts have been dissected and photographed with the same camera and microscope after grooming to evaluate the potential presence of pink powder.

The images of the eyes before and after grooming in intact and ablated insects were analyzed with the software ImageJ to evaluate the difference in areas contaminated with the powder before and after grooming in intact and ablated insects. The antennae of ten intact and ten ablated insects and the eyes of twelve intact and twelve ablated insects were analyzed. The percentage of contaminated area on the eyes of intact and ablated damselflies was compared using the Mann–Whitney Rank Sum Test.

## Results

### Morphology of the grooming devices

**Figure 1:**
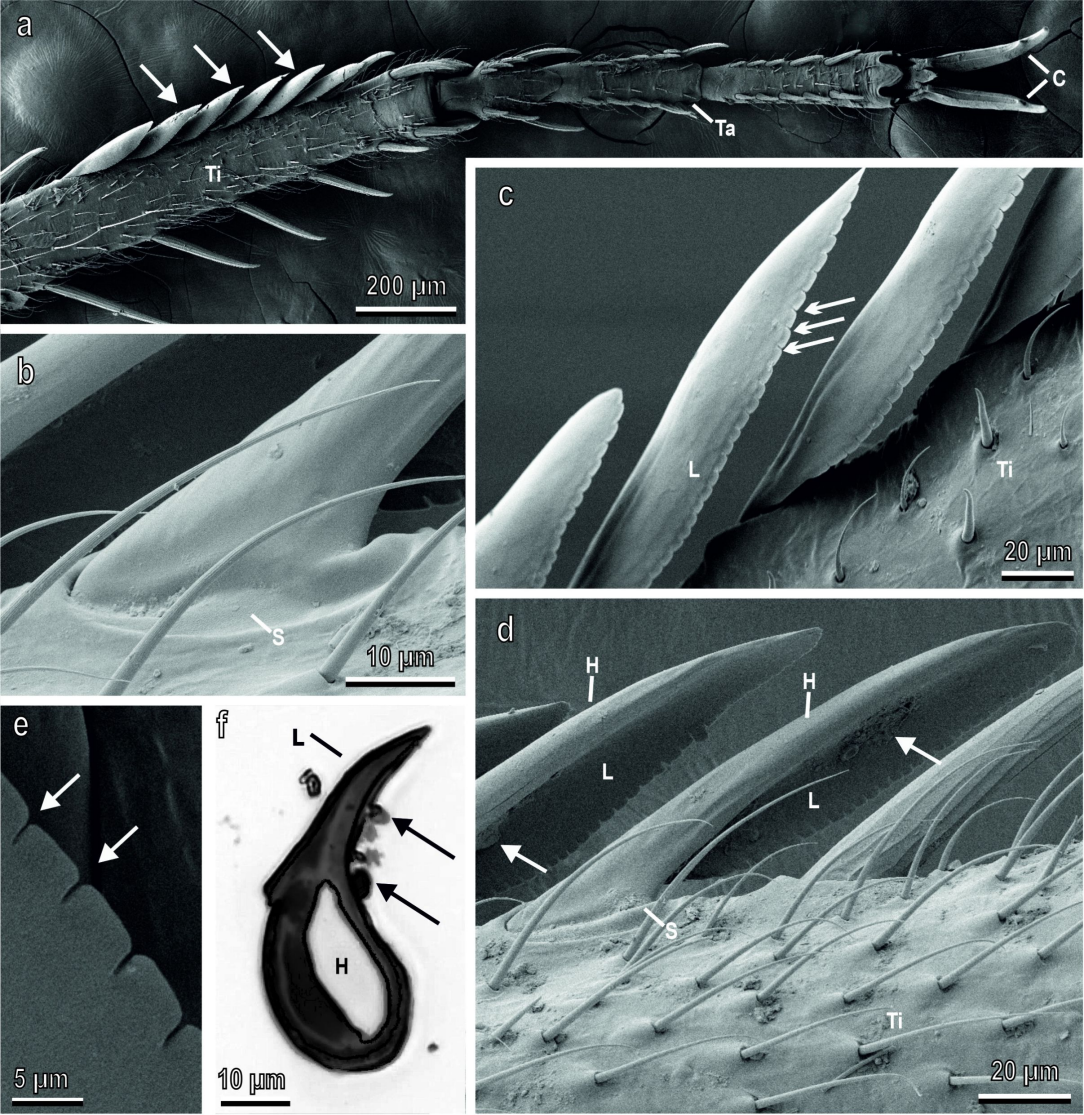
Left foreleg of *Ischnura elegans* (female) in SEM (a–e) and semithin section (f) of a grooming device under a light microscope. (a) Ventral view of tibia (Ti) and tarsus (Ta) showing the tibial grooming devices (arrows) located on the medial side of the tibia. C, claws. (b) Detail of a grooming device emerging from a well-developed socket (S). (c) Detail of the grooming devices observed from the ventral side of the leg. Note the border of the cuticular lamina (L) with indentations (arrows). (d) Grooming devices observed from the dorsal side of the tibia (Ti). Note that each grooming device is constituted of a long hair (H) from which a concave cuticular lamina (L) develops towards the medial side of the leg; arrows indicate the dirt particles accumulated inside the flag-shaped structures in correspondence of the concave cuticular lamina. S, socket. (e) Detail of the border of the cuticular lamina with indentations (arrows). (f) Cross section of a grooming device in its central portion. Note the hair (H) and the concave cuticular lamina (L) collecting dirt particles (arrows).

In the distal portion of the fore tibia of both sexes of *Ischnura elegans,* modified setae in the form of flag-shaped structures were visible (Figure 1a,c,d). They are located on the medial side of the tibia, and their number ranged from 7.08 ± 0.27 in males to 6.27 ± 0.24 in females. They measure about 210 µm in length and 44 µm in width and emerge from a well-developed socket (Figure 1b), which gives rise to a long seta, from which a concave cuticular lamina develops towards the medial side of the leg (Figure 1c,d). The border of the cuticular lamina showed indentations and appeared lobate along its medially oriented side (Figure 1c–e). In the cross section, the asymmetrical and concave shape of the grooming structures was clearly visible, with a thin lamina originating from a robust seta (Figure 1f). Dirt particles tended to accumulate inside the flag-shaped structures in correspondence with the concave cuticular lamina (Figure 1d,f). No sexual dimorphism has been observed regarding shape, size, and number of the grooming devices.

**Figure 2:**
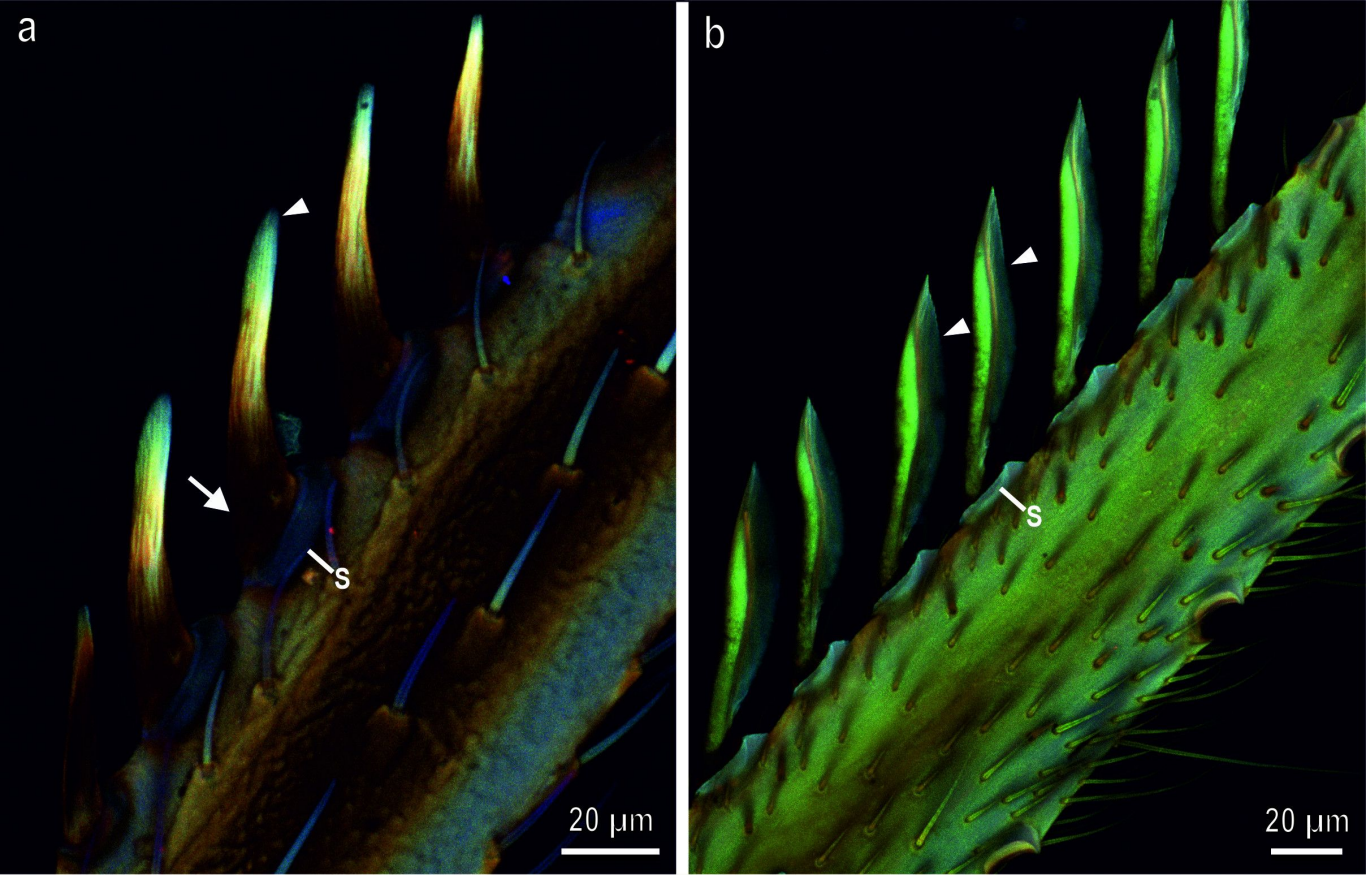
Confocal laser scanning micrographs (maximum intensity projections) showing differences in the autofluorescence composition present in the fore tibal grooming devices of *Ischnura elegans*. Red color indicates chitinous, sclerotized exoskeleton structures, green color indicates chitinous and non- or weakly sclerotized exoskeleton structures, and blue color indicates exoskeleton structures that either contain large proportions of the elastic protein resilin or are unsclerotized. (a) Dorsal view of the tibial grooming devices, revealing strongly sclerotized setae in their basal portion (arrow), which tend to become richer of resilin or unsclerotized and softer in their apical portions which are blue colored (arrow head). Note the soft setal socket (S), which is blue in color. (b) Ventral view of the tibial grooming devices. Note that the cuticular lamina appears blue (arrowhead) owing to either large proportions of resilin or its unsclerotized nature.

The CLSM analyses revealed that each tibial grooming device shows a different relative amount of resilin from its basal to its distal portion and from the hair to the cuticular lamina (Figure 2a,b). The flag-shaped grooming devices are set in a very soft socket, which appears blue, thus indicating a higher amount of resilin or unsclerotized chitin (Figure 2a). The base of the flag is very sclerotized in its basal portion appearing red and yellow, but tends to become richer in resilin or is unsclerotized in its apical portion, where more blue autofluorescence signal is visible (Figure 2a). The cuticular lamina appears blue, thus exhibiting large proportions of resilin or unsclerotized chitin (Figure 2b).

### Grooming behavior

**Figure 3:**
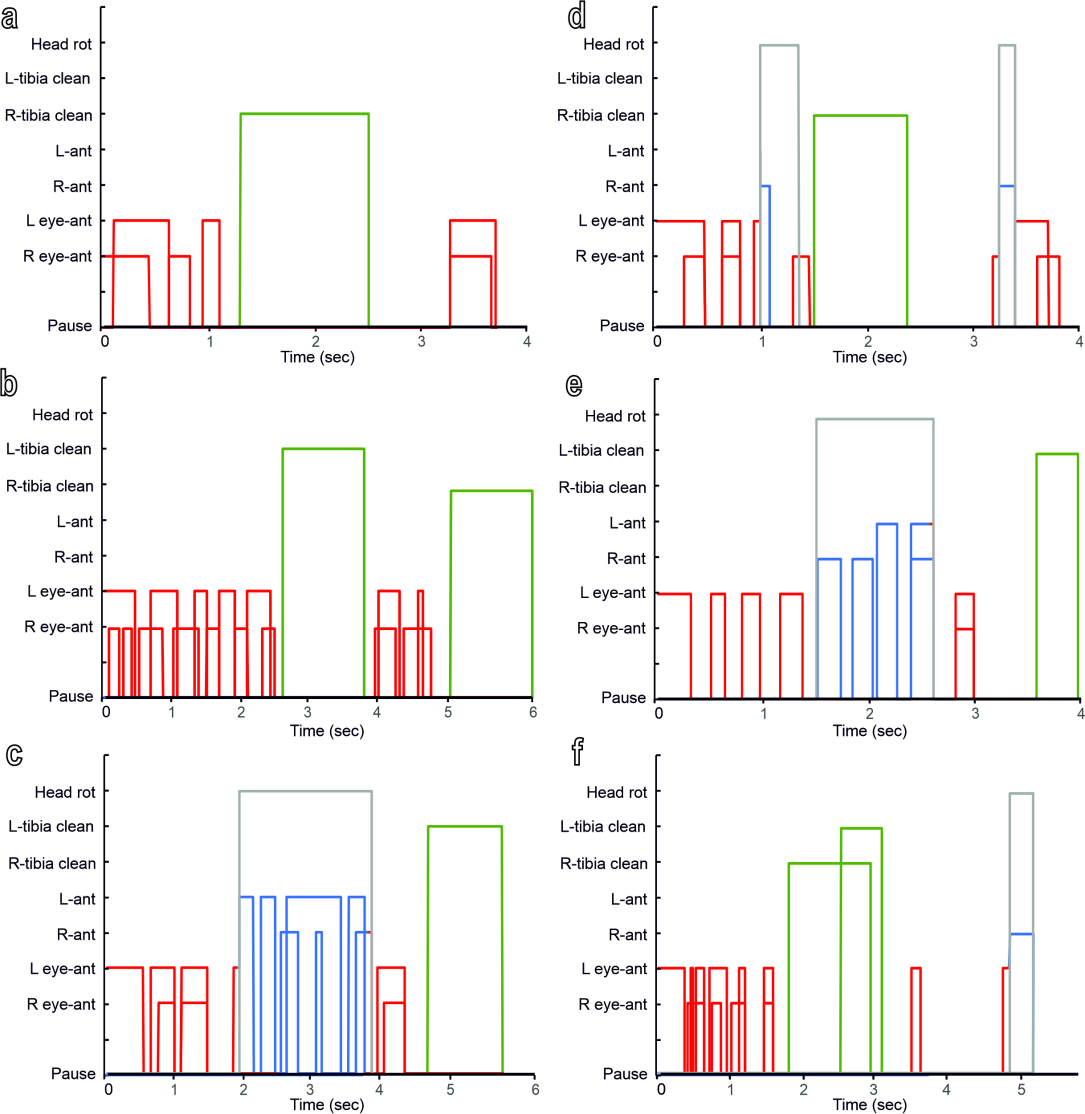
Ethograms of different bouts recorded during the grooming of *Ischnura elegans*. (a) First, (b), second and (c) fifth bout in the grooming of a damselfly with contaminated eyes. (d) First and (e) second bout in the grooming of a damselfly with contaminated antennae. (f) Single bout in the grooming of a damselfly with contaminated antennae. The following acts have been recorded: right foreleg raising and scraping of the right eye or of the right eye and of the dorsal side of the right antenna with the right tibia (R eye-ant); left foreleg raising and scraping of the left eye or of the left eye and of the dorsal side of the left antenna with the left tibia (L eye-ant); right foreleg raising and scraping of the right antenna with the right tibia (R ant); left foreleg raising and scraping of the left antenna with the left tibia (L ant); head rotation (70–90°) (head rot); right tibia running through mouthparts (R tibia cleaning); left tibia running through mouthparts (L tibia cleaning).

**Figure 4:**
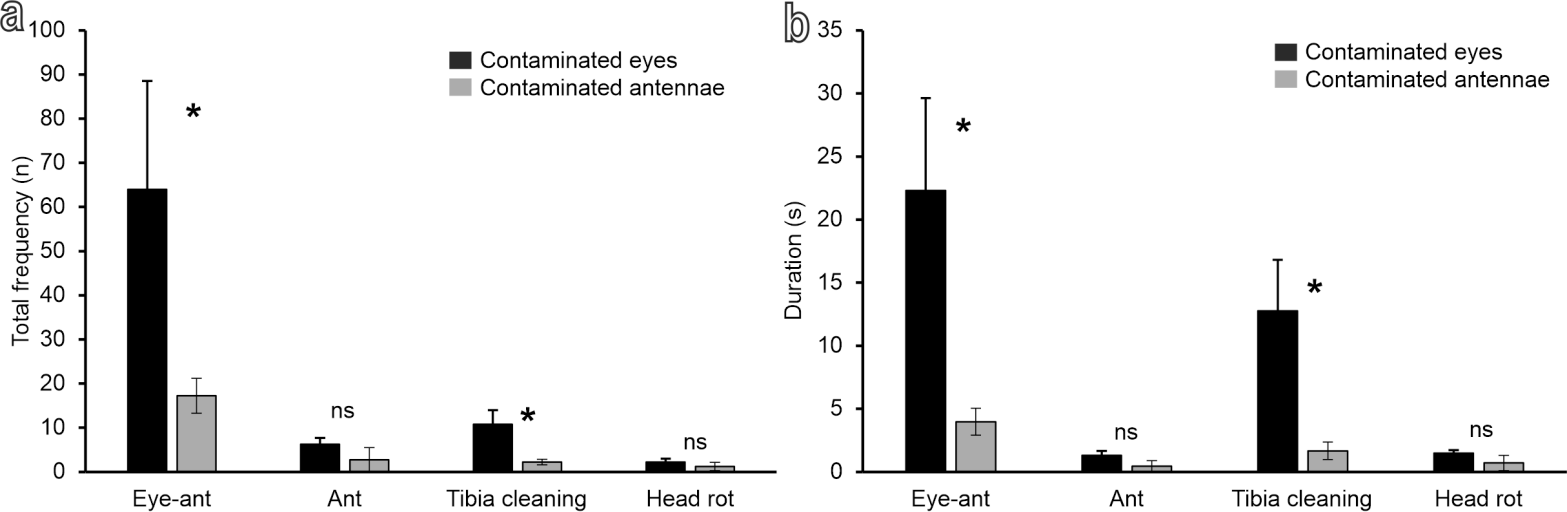
(a) Frequency and (b) duration of the different recorded acts during the grooming of *Ischnura elegans* in the experiments with contaminated eyes and with contaminated antennae. Forelegs raising and scraping of the eye(s) plus the dorsal side of the antennae with the tibiae (Eye-ant). Foreleg raising and scraping of the antennae with the tibiae (Ant). Tibia running through mouthparts (Tibia cleaning). Head rotation (70–90°) (Head rot). The asterisk indicates a significant difference at *p* < 0.05 (Mann–Whitney rank sum test for eye-ant and *t*-test for independent samples for the other acts).

The grooming of damselfly eyes and antennae occurred in bouts (Figure 3, Supporting Information File 1). The mean number of bouts before the insect rested for a time longer than 120 s was higher when the eyes were contaminated (4.3 ± 0.9) and lower (1.8 ± 0.5) when the antennae were contaminated (*t* = 2.6; d.f. = 6; *P* = 0.043). In each bout, damselflies performed a quick sequence of grooming acts, then stopped for a while before repeating a similar (but not the same) sequence of acts in a new bout (Figure 3). Damselflies cleaned their eyes even in the experiments with only antennal contamination and cleaned their antennae even in experiments with only eyes contaminated (Figure 3a–f). A sequence of acts of grooming eyes or antennae begins with one or both forelegs raising and scraping (a unidirectional movement from top to bottom) the ipsilateral eye and (afterwards) the ipsilateral antenna (R eye-ant and L eye-ant), with the tibial flag-shaped structures kept in contact first with the eye and afterwards with the dorsal side of the antenna. The action was repeated several times with both legs acting synchronously or with separate movements (Figure 3a–f). A series of eye and antennae scraping was almost always followed by dirt particle cleaning via the running of the tibial grooming structures through mouthparts (R and L tibia cleaning) (Figure 3a–f). Head rotation up to 90° was performed to allow antenna cleaning with the contralateral or ipsilateral tibia (R ant and L ant), in order to reach the side of the antennal surface previously not cleaned with the head in a horizontal position, thus improving the grooming efficiency (Figure 3). The frequency and duration of eye–antennal grooming (R eye-ant plus L eye-ant) (Frequency: *T* = 26; *N* = 8; *P* = 0.029. Duration: *T* = 26; *N* = 8; *P* = 0.029) and of tibia running through the mouthparts (R tibia cleaning plus L tibia cleaning) (Frequency: *t* = 2.57; d.f. = 6; *P* = 0.042. Duration: *t* = 2.69; d.f. = 6; *P* = 0.036) were higher in the experiments with contaminated eyes than in those with contaminated antennae, while there was no significant difference between the frequency (*t* = 1.13; d.f. = 6; *P* = 0.302) and duration (*t* = 1.48; d.f. = 6; *P* = 0.189) of antennal grooming (R ant plus L ant) in the experiments with contaminated eyes and in those with contaminated antennae (Figure 4a,b). There was no significant difference between the frequency (*t* = 0.83; d.f. = 6; *P* = 0.439) and duration (*t* = 1.17; d.f. = 6; *P* = 0.287) of head rotation (head rot) in the experiments with contaminated eyes and in those with contaminated antennae (Figure 4a,b). The mean duration of each bout was about 9.9 ± 2.6 s, when the eyes were contaminated and 4.7 ± 0.4 s, when the antennae were contaminated.

### Particle removal efficiency

**Figure 5:**
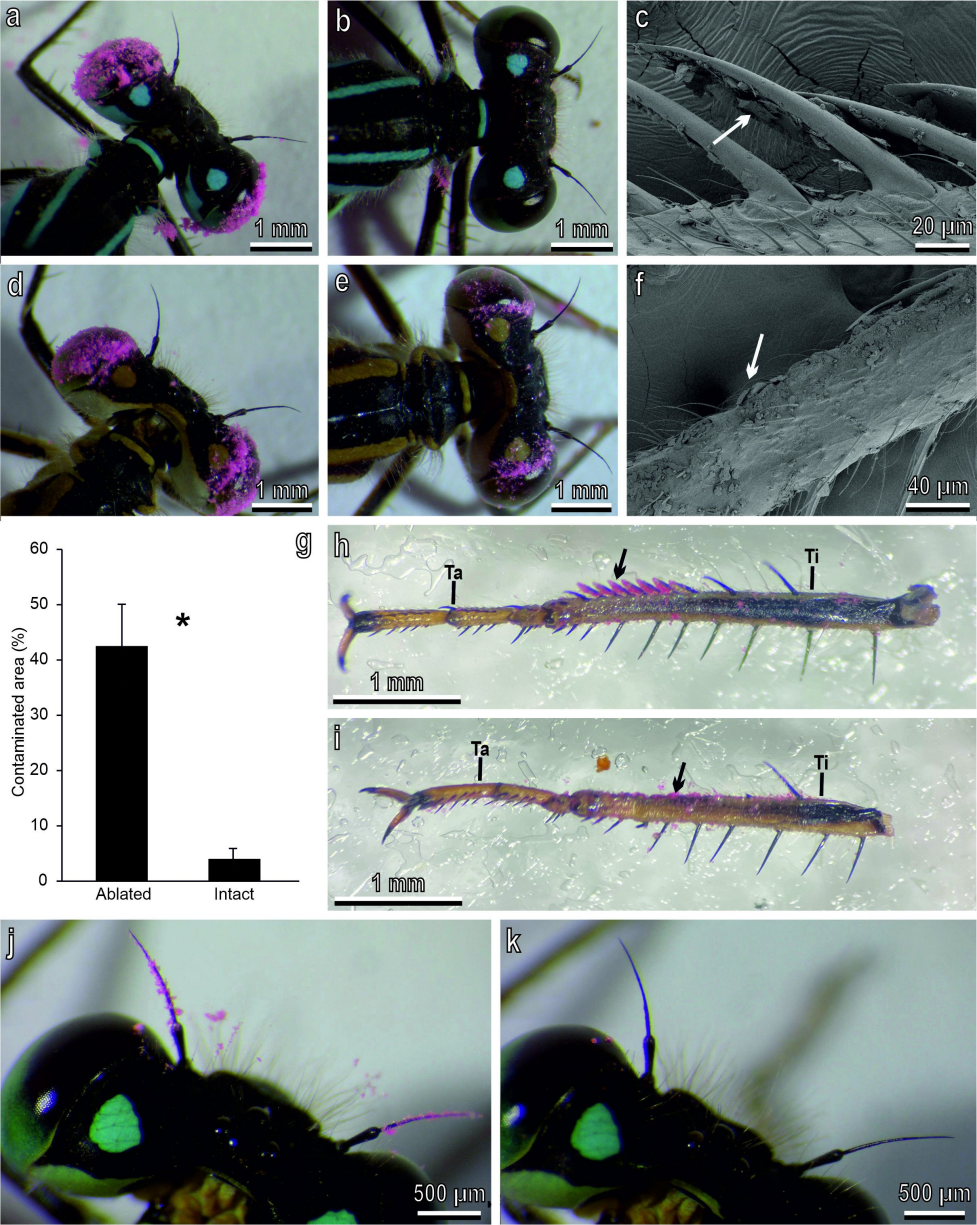
Particle removal efficiency in intact (a–c, h, j, k) and ablated (with the foretibial grooming structures artificially removed) (d–f, i) *Ischnura elegans* (female) under a stereomicroscope (a, b, d, e, h–k) and an SEM (c, f). (a, b) Intact damselflies with eyes contaminated by pink powder, just contaminated (a) and after one hour (b). Note the clean eyes. (c) Detail of the tibial grooming devices with pink powder (arrow) accumulated in correspondence of the concave side of the cuticular lamina. (d, e) Ablated damselflies with eyes contaminated by pink powder, just contaminated (d) and after one hour (e). Note the dirty eyes. (f) Detail of the tibia with the foretibial grooming structures artificially removed with powder (arrow) on the tibial surface. (g) Percentage of contaminated area on the eyes after cleaning of intact and ablated damselflies. The asterisk indicates a significant difference at *p* < 0.05 (Mann–Whitney rank sum test). (h) Tarsus (Ta) and tibia (Ti) with the tibial grooming devices (arrow) collecting pink powder. (i) Tarsus (Ta) and tibia (Ti) of an ablated damselfly. Note that some pink powder (arrow) is visible in correspondence of the grooming devices. (j, k) Intact damselfly with antennae contaminated by pink powder, just contaminated (j) and after (k) 10 min.

In the experiments, to evaluate the particle removal efficiency in intact (Figure 5a–c,h,j,k) and ablated (with their foretibial grooming structures artificially removed) (Figure 5d–f,i) insects, we observed that in intact damselflies the particle removal efficiency was very high leading to clean eyes (Figure 5b) and antennae (Figure 5k) after 1 h for the eyes and 10 min for the antennae. The pink powder removed from the eyes or antennae accumulated in correspondence with the flag-shaped structures located on the medial face of the tibia (Figure 5h), particularly in the concave side of the cuticular lamina (Figure 5c). The removal of the grooming devices in ablated insects (Figure 5f,i) induced a strong increase in the percentage of the areas contaminated with the powder on the eyes after grooming (Figure 5e). This percentage was 4.06% of the initial contaminated area in intact damselflies and 42.55% in ablated insects with a significant difference between the two values (*T* = 169; *N* = 22; *P* < 0.001) (Figure 5g).

**Figure 6:**
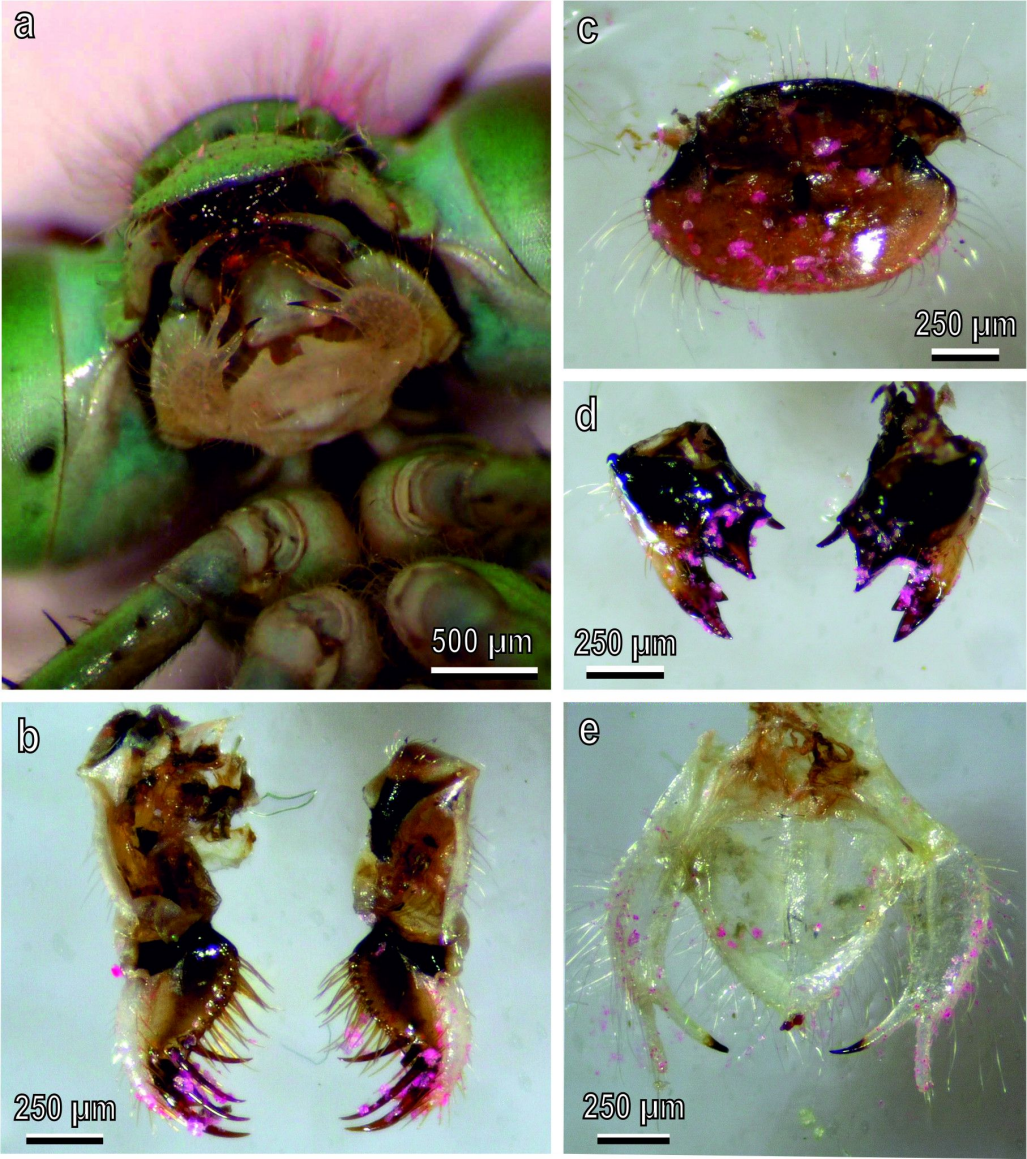
Mouthparts of *Ischnura elegans* dissected immediately after the antennal grooming behavior. (a) Ventral view of mouthparts with pink powder residues. (b) Dorsal view of the maxillae with evident pink powder, particularly on the maxillary palps. (c) Ventral view of the labrum with evident pink powder. (d) Dorsal view of the mandibles with some pink powder on the teeth. (e) Dorsal view of the labium with a reduced presence of pink powder.

In some intact specimens, after grooming, dissected mouthparts revealed the presence of pink powder (Figure 6a–e) visible in particular on the maxillae, especially on the maxillary palps (Figure 6a,b) and on the inner part of the labrum (Figure 6c). Some pink powder was also visible on the mandibular teeth (Figure 6d). The labium (Figure 6e) appeared less involved in tibial cleaning since the presence of pink powder appeared reduced in comparison with the other mouthparts.

## Discussion

Among the different micro- and nanostructures of the cuticular surface, grooming devices represent fundamental tools for insect survival. In insects with chewing mouthparts, such as Orthoptera, for instance Grylloblattodea, Dermaptera, Mantodea, and Blattodea, antennal grooming is typically performed using mouthparts to ingest debris [20,42]. These insects clean one antenna at a time by lowering it, then using the ipsilateral foreleg to pull it through the mouthparts from base to tip. This ancestral cleaning pattern has disappeared in insects with more specialized piercing-sucking or siphoning mouthparts, such as Hemiptera, Diptera and Lepidoptera, except the mandibulate archaic moth family Micropterigidae. Hemiptera use their forelegs to scrape their antennae, transferring debris onto the surrounding surface [20]. In contrast, many Hymenoptera employ a combination of techniques, using specialized leg spines to clean their antennae. They pass the antennae through a groove created by the apical tibial spur and the basitarsus of the foreleg, followed by using their mouthparts to clean the tibio-tarsal antenna cleaner [26–27,30,43].

Odonata possess biting mouthparts and ingestion of debris is the most likely strategy, but the behavior involving antennal cleaning with mouthparts cannot be performed owing to the short antennae. Odonata antennae in adults are reduced in size, but they possess different kind of sensilla, such as chemoreceptors [44–47] and thermo-hygroreceptors [44–48], which need frequent cleaning. Furthermore, the need to keep a clean eye surface to guarantee the functioning of the most advanced visual systems among insects ([35], review in [49]) requires the involvement of scraping forelegs with specialized cleaning structures. In our experiments, we could observe a higher frequency and a higher duration of grooming acts when the eyes were contaminated in comparison with antennal contamination, but this is probably due to the higher amount of powder used to contaminate eyes in comparison with that used for antennal contamination. Special attention is devoted by damselflies to their antennae. In particular, they rotate the head up to 90° to allow antenna cleaning with the contralateral or ipsilateral tibia, in order to reach the side of the antennal surface not previously cleaned with the head in horizontal position, thus improving the grooming efficiency. In any case, we could observe that, in each bout, antennae and eyes are always cleaned together, exactly as observed in *Drosophila* where antennal grooming is elicited via mechanoreceptors of Johnston’s organ [50]; when legs of *Drosophila* sweep across the antennae, they also sweep across the eyes.

Antennal grooming organs have independently evolved in several insect orders, differing in the morphology and surface complexity of the involved structures [51–52]. These cleaning organs typically consist of modified setae and other cuticular projections that scrape and remove particles, concentrating them for disposal [53]. In *Ischnura elegans*, grooming devices include setae with concave cuticular laminae on the medial side of the tibiae. Each seta emerges from a soft socket either rich in resilin or of unsclerotized chitin, which enables movement at the base, proceeds in a hard base of sclerotized chitin, and ends in a soft tip, either rich in resilin or of unsclerotized chitin. A material gradient from stiff bases to soft tips has also been found in the adhesive hairs of insect leg attachment systems [38–39,54]. This gradient prevents the clustering of adhesive hairs, while the soft tips ensure effective contact between the attachment system and the substrate. Similarly, the material gradient in grooming devices may enhance adhesion to foreign materials for grooming body surfaces. The soft tip and soft lateral cuticular lamina adapt to various surface geometries, while the stiff base prevents clumping of setae. The elasticity of the cuticular lamina of the grooming devices of *I. elegans* enhances the ability of the grooming structures to scrape eyes and antennae removing and collecting debris that accumulated inside the flag-shaped structures. The indentations along the lateral border of the lamina can further help in entrapping dirt particles.

The effectiveness of these structures in Odonata grooming is clearly demonstrated in our experiments with ablated insects, where we can observe a strong increase in the percentage of the contaminated areas on the eyes after grooming. In different insect orders, grooming devices are represented by modified setae from which thin cuticular laminae develop on one or on both lateral sides. This is the case of Mantodea, where a femoral brush comprising 100–200 feather- or paddle-shaped setae is present [32]. In Hemiptera, the tibial comb is constituted of a concave cuticular lamina, whose distal border bears a row of tightly packed digitiform setae with lateral cuticular laminar expansions, which overlap allowing the interdigitations of the setae [28]. The digitiform setae have a gradient of resilin concentration and, therefore, mechanical properties [28]. The interdigitated cuticular laminar expansions overlapping at different heights constitute a very flexible surface because of their high resilin content, which enables them to gently press against the antennal surface to be cleaned, thereby squeezing the debris outwards [28]. Many Hymenoptera bear flattened leg spines specialized for the cleaning of antennae [26–27,55]. A high content of resilin characterizes the inner side of the calcar or the basitarsal comb in the antenna cleaner of different species of Formicidae [56]. Zhang et al. [33], through nanoindentation tests, discovered that the tibial comb of honeybees exhibits a resilin gradient with a stiffness variation spanning nearly two orders of magnitude, ranging from approximately 25 MPa at the tip to around 645 MPa at the base. This gradient enhances the catapult effect, allowing the comb to produce increased inertia that counteracts the initially dominant adhesion, effectively dislodging attached pollen and dust. The same authors also developed an elastomeric bioinspired stiffness-gradient catapult and demonstrated its potential in practical applications, thus confirming that studies on the functional morphology of insect grooming devices can represent a starting point for further investigation of optimal materials design in bioinspired robotic systems.

As observed in our experiments, Odonata clean their grooming devices running their forelegs through the mouthparts. In our observations, we could not detect a special mouthpart involved in collecting particles from the grooming devices since the pink powder can be observed in the distal portion of maxillae, especially on the dentisetae, which are very sclerotized [57], in the mandibles, and in the inner portion of the labrum. In the German cockroach, which possesses chewing mouthparts and achieves antennal debris removal scraping directly the antenna over the glossa, most of the debris are manipulated into the hypopharynx and ingested when grooming is completed [42]. Further studies could clarify the biomechanics of foretibiae cleaning in Odonata. In this regard, it is important to remember that understanding insect grooming may provide insights into routes of entry of pesticides because the oral toxicity of substances that induce grooming (such as insecticides or other chemicals toxic for insects) should increase in insects that include ingestion in their grooming behavior [2].

As reported above, grooming behavior and devices, especially in insects, have been used to investigate phylogeny and evolution because of their low variability within species and relative evolutionary conservatism [26,30,52]. Further investigation of the grooming devices of different Odonata families, which diverged relatively early in their evolution, especially in relation to morphology of their compound eyes, would be interesting in this context.

## Supporting Information

File 1 Damselflies’ antennal and eye grooming behavior.

## Data Availability

The data that supports the findings of this study is available from the corresponding author upon reasonable request.
